# Pathology Foundation Models: Evolution, Current Landscape, Challenges and Opportunities from a Technical and Clinical Perspective

**DOI:** 10.3390/bioengineering13050577

**Published:** 2026-05-19

**Authors:** Hussien Al-Asi, Ibrahim Yilmaz, Jordan Reynolds, Shweta Agarwal, Aziza Nassar, Abba Zubair, Craig Horbinski, Bryan Dangott, Zeynettin Akkus

**Affiliations:** 1 Department of Laboratory Medicine and Pathology, Mayo Clinic Florida, 4500 San Pablo Rd S, Jacksonville, FL 32224, USA; alasi.hussien@mayo.edu (H.A.-A.); yilmaz.ibrahim@mayo.edu (I.Y.); reynolds.jordan@mayo.edu (J.R.); agarwal.shweta@mayo.edu (S.A.); nassar.aziza@mayo.edu (A.N.); zubair.abba@mayo.edu (A.Z.); horbinski.craig@mayo.edu (C.H.); dangott.bryan@mayo.edu (B.D.); 2 Computational Pathology and AI/Informatics, Department of Laboratory Medicine and Pathology, Mayo Clinic Florida, 4500 San Pablo Rd S, Jacksonville, FL 32224, USA; 3 Department of Artificial Intelligence and Informatics, Mayo Clinic Florida, 4500 San Pablo Rd S, Jacksonville, FL 32224, USA

**Keywords:** pathology foundation models, vision encoders, vision transformers, deep learning, artificial intelligence

## Abstract

Foundation models are reshaping computational pathology by enabling scalable task-agnostic representations of histopathological whole-slide images (WSIs). Unlike earlier task-specific deep learning systems, pathology foundation models (PFMs) leverage massive whole-slide image repositories and self-supervised Vision Transformer architectures to achieve broad generalization and few-shot adaptability. Their evolution reflects a shift from weakly supervised approaches such as Clustering-Constrained Attention Multiple Instance Learning (CLAM) and hierarchical architectures such as Hierarchical Image Pyramid Transformer (HIPT) to large-scale efforts including foundation models, UNI, Virchow, Phikon, CONtrastive learning from Captions for Histopathology (CONCH), GigaPath, H-Optimus, Transformer-Based Pathology Image and Text Alignment Network (TITAN), and the Mayo Clinic Atlas. These models demonstrate impressive performance across diagnostic and prognostic benchmarks while also opening pathways for multimodal integration with genomics and clinical data. Yet significant barriers remain including inconsistent generalization across institutions, interpretability lagging behind clinical needs, and slow integration into routine laboratory workflows. Certain domains of anatomic pathology such as cytopathology, transplant pathology, frozen sections, and rare tumor subtypes remain particularly resistant to current models. Here, we review the development of PFMs, critically evaluate their strengths and limitations, and outline priorities for their safe and effective clinical translation. We argue that the next phase of PFM development will depend on rigorous benchmarking, pathologist-in-the-loop deployment, and multimodal fusion ensuring these models evolve from research tools into clinically robust systems.

## 1. Introduction

The emergence of attention mechanisms has fundamentally reshaped modern computational modeling by enabling systems to selectively emphasize informative components within complex inputs [1]. This paradigm shift culminated in the development of transformer architectures, which rapidly became the foundation for scalable and highly expansive models across multiple domains [2].

**Figure 1:**
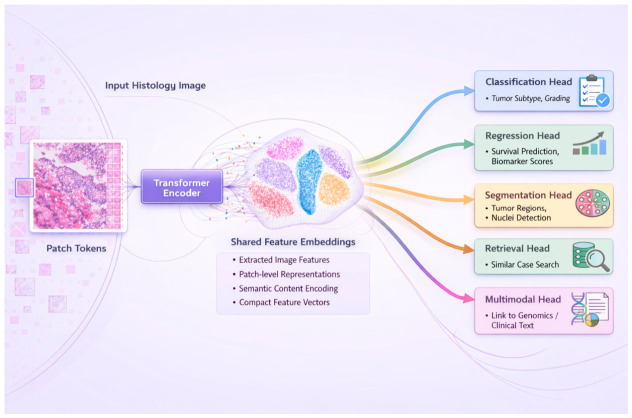
Vision Transformer-based multi-task pathology framework. Histology images are tokenized and encoded into a shared embedding, which supports multiple downstream tasks including classification, regression, segmentation, retrieval, and multimodal integration.

In natural language processing, transformer architectures directly enabled the exponential growth of large language models, demonstrating that self-attention could capture long-range contextual dependencies without reliance on handcrafted features [3]. Parallel efforts in computer vision applied similar principles by representing images as sequences of embedded patches, giving rise to Vision Transformer architectures capable of global spatial reasoning (Figure 1) [4].

Furthermore, the application of Vision Transformers to histopathology has driven the development of PFMs, designed to learn generalizable morphologic representations from WSIs. These models mark a departure from task-specific pipelines toward reusable task-agnostic embeddings that can support a wide range of diagnostic and prognostic applications [5,6,7].

Earlier computational pathology approaches relied predominantly on manually trained and fine-tuned convolutional neural networks including architectures such as ResNet, DenseNet, and U-Net [8,9,10,11]. While effective for narrowly defined tasks such as tumor detection and segmentation, these models required extensive annotation and demonstrated limited robustness across institutions and disease contexts [11,12,13,14,15,16].

Subsequent architectural advances addressed data efficiency and scalability. Model architectures such as Data-Efficient Image Transformers (DeiTs), Shifted Window Transformers (Swin), and Hierarchical Image Pyramid Transformer (HIPT) in addition to techniques such as masked autoencoding introduced hierarchical and window-based attention mechanisms suitable for high-resolution imagery [17,18,19]. In contrast, segmentation methods evolved from convolutional frameworks to more flexible approaches including promptable segmentation models such as the Segment Anything Model (SAM) [20,21].

As pathology datasets expanded to include hundreds of thousands of WSIs, scalable solutions for image parsing, indexing, and retrieval became necessary. Early systems enabled large-scale similarity search but focused on limited objectives [22,23,24]. This was followed by a landscape shift towards general-purpose PFMs centered around novel transformer architectures. In addition, these foundation models boasted hundreds of thousands (e.g., UNI, GigaPath and Phikon) up to millions of WSIs (e.g., Virchow, Virchow 2.0, Mayo Atlas, and Transformer-Based Pathology Image and Text Alignment Network (TITAN)) [25,26,27,28,29,30,31]. The timeline of these developments illustrates how fast they have occurred (Table 1).

This review examines pathology foundation models through a clinical lens with a focus on unmet diagnostic needs, current implementations, barriers to clinical adoption, known pitfalls, the state of benchmarking, and future directions required for safe and effective integration into anatomic pathology practice.

A structured literature search was conducted in PubMed using the following query: ((“large language model” OR “LLM” OR “foundation model” OR “transformer model” OR “GPT” OR “BERT” OR “language model”) AND (“pathology” OR “computational pathology” OR “digital pathology” OR “histopathology” OR “anatomic pathology”)). The following terms used in the search query represent (LLM and BERT) represent large language models and Bidirectional Encoder Representations from Transformers respectively. Furthermore, the query aimed at ensuring there was equal representation between vision-only and vision–language models included in the review.

**Figure 2:**
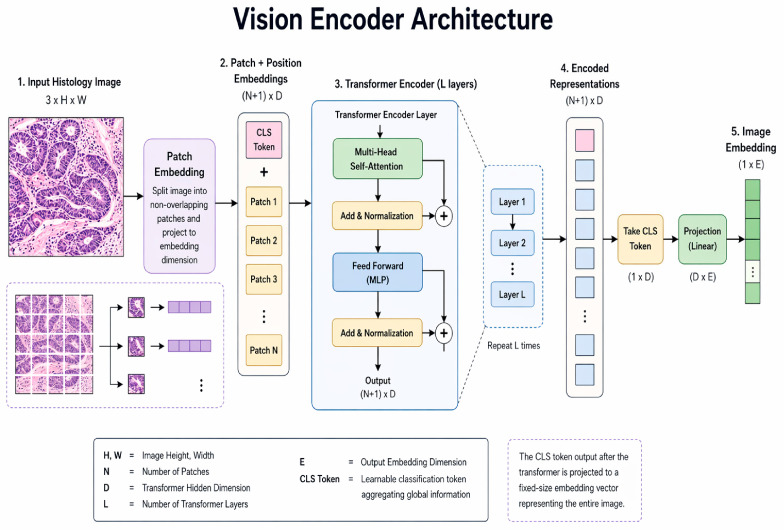
Vision encoder architecture for histology image. Schematic of the Vision Transformer encoder architecture applied to whole-slide histopathology images, highlighting patch embedding and stacked transformer blocks. Embedded image patches are processed through multi-head self-attention, multiple-instance learning, and feed-forward layers to model long-range contextual relationships. The encoder produces both global and patch-level representations that serve as inputs for downstream pathology tasks.

In recognition of the rapid evolution of computational pathology, relevant preprints were also considered as many state-of-the-art models are disseminated prior to peer review and are frequently accompanied by publicly available code. Inclusion of these sources was limited to studies with sufficient methodological transparency to allow critical appraisal. Furthermore, inclusion criteria for the included studies ranged from 2020 to 2025 for transformer-based models only. Figure 2 represents the basic structure of Vision Transformer models included in this review [4,5]. The exclusion criterion was papers on models that were based solely on convolutional neural network architectures.

## 2. Clinical Motivation and Diagnostic Context

Contemporary anatomic pathology is characterized by steadily increasing case volumes accompanied by a growing reliance on ancillary studies. Routine surgical pathology specimens are now frequently supplemented by immunohistochemistry, in situ hybridization, molecular assays, and increasingly complex reflex testing algorithms [32]. While these modalities enhance diagnostic precision, they also increase interpretive burden, turnaround time, and cognitive load for pathologists, particularly in high-throughput academic reference laboratory settings and governmental laboratories [33,34].

In parallel, the diagnostic complexity of individual specimens has increased. Many cases now require integration of subtle morphologic features with immunophenotypic and molecular findings often within evolving disease classification frameworks [35,36]. The rapid expansion of molecular testing has further transformed the practice of anatomic pathology, shifting the pathologist’s role from purely morphologic assessment toward synthesis of multimodal data [37,38]. This evolution has introduced new challenges in consistency, reproducibility, and decision-making, particularly in borderline or heterogeneous lesions [39,40].

Within this context, task-specific computational pathology approaches have demonstrated value in narrowly defined scenarios, particularly where automated triage or detection can reduce time to diagnosis [25]. However, these approaches often fail to generalize beyond their intended use cases [25]. In contrast, so-called “catch-all” foundation models have thus far shown more limited success in routine practice, reflecting the highly variable, context-dependent, and institution-specific nature of histopathology [41]. The diversity of tissue types, preparation artifacts, and diagnostic objectives presents a fundamental challenge to models that aim to provide universal representations without sufficient clinical grounding [42].

## 3. Current Implementations of PFMs

Over the past several years, multiple PFMs have been released by both academic and industry teams, reflecting growing interest in scalable and generalizable approaches to histopathology analysis [21,26,27,28,29,30,31,43,44,45,46]. These models vary in architectural design, training strategy, and intended scope, ranging from open academic encoders to institutionally developed systems. Table 2 illustrates a breakdown of the chronological evolution of prominent PFMs as well as model enabling architecture.

Several studies have evaluated the performance of these models across a range of downstream tasks including cancer classification, subtype prediction, biomarker inference, and prognostic modeling [42,47,48,49]. These comparative evaluations offer valuable insight into model capabilities within specific experimental contexts. However, such assessments are often conducted using task suites developed by the investigative teams themselves rather than standardized or externally validated benchmarks [50]. While this approach allows models to be tested on clinically motivated use cases, it complicates direct comparison across studies and raises questions regarding generalizability [51]. Performance gains demonstrated within narrowly defined task sets may not translate to broader clinical practice, particularly when applied across institutions, tissue types, or specimen preparations [52]. This heterogeneity in evaluation highlights an important gap between reported performance and real-world deployment, underscoring the need for more consistent benchmarking frameworks in computational pathology [52].

## 4. Performance and Benchmarking Landscape

The Cancer Genome Atlas (TCGA) with approximately 29,000 whole-slide images (WSIs) remains the most widely used public histopathology dataset. However, despite its utility, TCGA is heavily biased toward patients of European ancestry (>80%) and under-represents Black, Asian, and Hispanic populations [53,54]. Moreover, public datasets such as CAMELYON16 [55] often exhibit site-specific artifacts including staining variations or pen marks, which models can exploit as shortcuts rather than learning true biological features [56]. To address these limitations, newer benchmarks have been developed. These include PathoBench, which provides standardized train–test splits across 42 clinically relevant tasks, PathBench, which covers 64 tasks across 10 hospitals with private data to prevent pretraining leakage, and MEDFAIR, which evaluates algorithmic fairness across diverse datasets [54,57].

These frameworks provide canonical data splits and evaluation protocols for over 60 clinically relevant tasks including morphological subtyping, molecular biomarker prediction, and survival prognosis [54,57]. These initiatives are critical for ensuring transparency and preventing data leakage, which occurs when evaluation data is inadvertently included in the massive pretraining corpus of a model [47,57].

Performance evaluations across these benchmarks consistently identify Virchow2, CONCH, and H-Optimus-1 as the top-tier models currently available [54,57]. CONCH, a vision–language model, frequently demonstrates superior generalizability in predicting complex biomarkers such as microsatellite instability (MSI) due to its multimodal pretraining which aligns visual features with textual clinical descriptors [47,57]. Meanwhile, vision-only models such as Virchow2 and H-Optimus-1 lead in histological subtyping and tumor grading across diverse organs including breast, gastric, and colorectal cancers [57]. A key insight from this data is that pretraining data diversity, the variety of anatomical sites and cancer types, often outweighs sheer data volume in determining the downstream success of a model downstream success [55,57]. Adapting these massive foundation models to specific clinical tasks requires efficient fine-tuning strategies as evidenced by benchmarking studies (PathBench, PathoBench, and Trident) indicating that Parameter-Efficient Fine-Tuning (PEFT) such as Low-Rank Adaptation (LoRA) is significantly more effective than traditional linear probing, offering high accuracy while minimizing computational costs [57]. Moreover, in data-constrained scenarios where only a few examples are available, few-shot learning methods that modify the model during the testing phase only such as Baseline and Baseline++ have proven most effective [58,59,60,61,62,63,64]. While histopathology has seen the most rapid advancement, cytopathology is beginning to integrate these technologies. Furthermore, deep learning models have already achieved diagnostic accuracies up to 96% in breast and thyroid cytology studies, despite unique challenges such as the need for 3D Z-stacking to capture thick cell clusters [58,59].

On the other hand, many academic AI studies focus on strongly supervised tasks such as subtyping or grading, while clinical practice increasingly demands weakly supervised insights including genetic alterations, treatment response, and survival prediction [60]. Compounding this issue, real-world WSIs are gigapixel-scale, yet many models are trained on small patches or tissue microarrays, which fail to capture the spatial heterogeneity of a full slide [48,49]. Additionally, evolving clinical criteria such as updates to the International Classification of Diseases (ICD) introduce “concept drift” [61] and many AI products offer only incremental improvements, limiting their practical adoption despite high development costs [54].

Understanding concept drift in the context of PFMs provides a technical solution for diagnosing “black box” failures by identifying whether a performance drop is caused by technical domain shifts such as varying stain protocols and scanner artifacts or fundamental shifts in clinical diagnostic criteria [61,62]. By leveraging global feature importance, researchers can isolate the specific morphological markers driving the drift while counterfactual explanations can visually demonstrate to a pathologist how features of a tissue sample would need to be modified to alter the diagnostic outcome of a model [63], ultimately fostering the trust and acceptance required for deploying adaptive AI systems in routine clinical practice [64].

As a result, models often fail to generalize across institutions due to distribution shifts in fixation, staining, and scanner parameters [56]. Furthermore, performance can be artificially inflated by data leakage from pretraining corpora and most evaluations lack demographic or institutional stratification [56]. Therefore, robust clinical validation requires independent datasets spanning multiple sites and populations to ensure that models are genuinely “fit for purpose” across diverse clinical contexts [65].

## 5. Pitfalls and Failure Modes in Practice for Subspecialty Domains

Despite growing interest in clinical deployment, pathology AI systems continue to encounter recurring failure modes that limit real-world utility, particularly outside solid tumor histopathology [60,65,66]. These challenges are most evident in cytopathology and hematopathology, where differences in specimen preparation, diagnostic workflows, and interpretive complexity expose limitations not fully captured in benchmark studies [58,62].

Operational barriers remain substantial. High costs associated with whole-slide scanners and long-term storage of gigapixel whole-slide images constrain adoption while cytology-specific requirements such as Z-stacking for thick preparations introduce additional technical complexity and artifact susceptibility [58,59]. At the model level, many systems are trained on limited retrospective datasets, leading to overfitting and poor external performance [58,59]. The scarcity of prospective multi-institutional validation further complicates routine clinical integration.

Subspecialty blind spots also persist. AI development has largely focused on common entities with rare diseases and hematopathology remaining under-represented due to data imbalance and diagnostic heterogeneity [48]. Even large foundation models may disproportionately encode dominant organ systems, limiting their effectiveness in less common but clinically critical contexts [63].

Finally, domain shift and misalignment with pathologist reasoning remain central challenges. Variations in staining, scanning, and preparation across laboratories can significantly alter model behavior while nonbiologic artifacts may be inadvertently learned as diagnostic cues [54]. In addition, many models rely on abstract feature representations that do not map cleanly onto established morphologic criteria, limiting interpretability and trust [56,65]. Together, these limitations underscore the gap between experimental performance and clinical readiness and motivate the need for more deployment-aware evaluation and design strategies as discussed below.

Studies have demonstrated that single-plane imaging may be insufficient for diagnostic adequacy in cytopathology, particularly in liquid-based preparations where Z-stacking is required to capture the three-dimensional architecture of cellular clusters [67]. In addition, this is mirrored in thyroid cytology where a single focal plane may fail to encompass diagnostically relevant features [68]. These findings highlight a fundamental limitation in current digital pathology pipelines as most foundation models are trained on single-plane WSIs. Consequently, cytologic specimens such as smears and aspirates, characterized by variable thickness and three-dimensional cellular organization, remain under-represented and poorly modeled, posing a significant barrier to the deployment of AI in digital cytology workflows [69].

In parallel, the diagnosis of hematologic disorders relies heavily on multimodal ancillary studies including flow cytometry, cytogenetics, and molecular assays [66]. These data modalities are integral to clinical decision-making but are largely absent from the datasets used to train current PFMs [58,66]. This lack of multimodal context combined with the relative under-representation of hematopathology in training cohorts likely contributes to reduced model performance in this domain, particularly for diagnostically complex or rare entities [58,63].

## 6. Barriers to Clinical Adoption

Despite rapid technical progress, multiple barriers continue to limit the translation of PFMs into routine clinical practice. Regulatory and validation challenges remain a primary constraint. Unlike conventional software, PFMs are inherently adaptive and often repurposed across tasks, complicating regulatory classification and approval pathways [60]. Current frameworks (e.g., Food and Drug Administration’s (FDA) Software as a Medical Device (SaMD)) are largely designed for fixed-function algorithms and do not yet fully accommodate continuously learning or task-agnostic systems [54]. Furthermore, most PFMs lack prospective multi-site clinical validation studies required to demonstrate safety and effectiveness in real-world workflows [60].

Infrastructure and deployment complexity also present significant obstacles. Whole-slide image storage, retrieval, and processing require substantial computational resources including high-performance graphical processing units (GPUs), scalable storage systems, and low-latency networking [69]. Integration with laboratory information systems (LISs), digital pathology viewers, and enterprise authentication frameworks (e.g., PACS, cloud environments, secure APIs) introduces additional engineering and security burdens that extend beyond model development [70].

Workflow integration and human factors are equally critical. Pathology workflows are highly structured and time-sensitive and introducing AI systems without disrupting efficiency or diagnostic confidence remains challenging [54,60]. Models that operate as “black boxes” without interpretable outputs are unlikely to gain pathologist trust. Moreover, pathologist–AI interaction paradigms are still evolving with limited consensus on optimal interfaces, feedback mechanisms, or escalation pathways in cases of disagreement [60].

Data governance, privacy, and ownership further complicate adoption. Large-scale model training often requires aggregation of multi-institutional datasets, raising concerns regarding patient privacy, data sharing agreements, and institutional control [56]. In addition, variability in data quality, annotation standards, and metadata completeness can limit reproducibility and model robustness [54].

Economic and reimbursement considerations also play a key role. The cost of digital pathology infrastructure combined with uncertain reimbursement pathways for AI-assisted diagnostics creates a barrier to adoption, particularly in smaller institutions [60,71], demonstrating clear clinical utility, cost-effectiveness, and return on investment will be essential for widespread deployment.

Together, these factors highlight that the primary bottlenecks are no longer purely algorithmic, but rather systemic, requiring coordinated advances in regulation, infrastructure, workflow design, and clinical validation.

## 7. Future Directions and Clinical Translation

The next phase of PFM development will depend on a shift from model-centric innovation toward deployment-centric design and evaluation.

### 7.1. Standardized Clinically Meaningful Benchmarking

Future efforts must prioritize externally validated multi-institutional benchmarks that reflect real-world variability in specimen preparation, scanner platforms, and patient populations [48]. Benchmarking should extend beyond classification accuracy to include clinically relevant endpoints such as diagnostic concordance, time savings, and impact on patient management.

### 7.2. Pathologist-in-the-Loop Systems

Rather than fully autonomous systems, PFMs are likely to achieve the greatest impact as decision-support tools. Interactive frameworks that allow pathologists to query, refine, and validate model outputs, potentially through retrieval-augmented or conversational interfaces, can improve both usability and trust. These systems should support iterative feedback and continuous learning within controlled governance frameworks [72].

### 7.3. Future Iterations

Models must integrate histopathology with complementary data modalities including genomics, radiology, laboratory values, and clinical notes. Vision–language models and multimodal transformers offer a promising pathway toward more comprehensive disease modeling, aligning more closely with real-world diagnostic reasoning [31].

### 7.4. Domain-Specific Specialization Within General Frameworks

While general-purpose PFMs provide broad representations, hybrid approaches that combine foundation models with domain-specific fine-tuning may be necessary for challenging areas such as cytopathology, transplant pathology, and hematopathology. This includes incorporation of 3D imaging (e.g., Z-stacks), temporal data, and rare disease cohorts [29,66,69,73].

### 7.5. Robustness to Domain Shift and Artifact Awareness

Future systems must explicitly model and mitigate domain shift including staining variation, scanner differences, and preparation artifacts. Techniques such as stain normalization, domain adaptation, and uncertainty quantification will be critical for safe deployment [52].

### 7.6. Interpretability and Alignment with Pathology Ontology

Interpretability must evolve beyond attention maps toward clinically meaningful explanations that align with established morphologic criteria and diagnostic frameworks. Linking model outputs to recognizable histologic features (e.g., nuclear atypia and architectural patterns) will be essential for adoption [64].

### 7.7. Prospective Clinical Trials and Real-World Validation

Ultimately, the transition to clinical practice will require prospective, workflow-integrated studies demonstrating improved diagnostic accuracy, efficiency, or patient outcomes. These studies should evaluate performance across diverse institutions and include human–AI interaction metrics [74].

## 8. Conclusions

PFMs represent a significant conceptual and technical shift in computational pathology, moving the field from narrowly trained task-specific algorithms toward scalable and reusable representations of histopathology. These models have demonstrated promising performance across a range of diagnostic, prognostic, and predictive tasks while enabling new capabilities such as cross-task transfer, multimodal integration, and large-scale image retrieval. In addition, they offer the potential to unify disparate analytical pipelines under a common representational framework, reducing redundancy and enabling more efficient development of downstream applications.

Despite these advances, the translation of PFMs into routine clinical practice remains limited. Current models are often evaluated in controlled experimental settings that do not fully capture the variability, complexity, and workflow constraints of real-world pathology. Differences in tissue processing, staining protocols, and scanner platforms introduce variability that can significantly impact model performance. Challenges related to generalizability, benchmarking, interpretability, and domain-specific performance, particularly in areas such as cytopathology, hematopathology, and transplant pathology, continue to limit their clinical utility. Furthermore, inconsistencies in evaluation frameworks and the absence of standardized benchmarks make it difficult to directly compare models or assess readiness for deployment.

Importantly, the primary barriers to adoption are no longer purely algorithmic. Instead, they reflect broader system-level challenges including integration with laboratory infrastructure, regulatory pathways, data governance, and alignment with pathologist workflows. Issues such as interoperability with laboratory information systems, data privacy constraints, and the need for continuous model monitoring further complicate implementation. Addressing these challenges will require a shift from model-centric innovation toward deployment-aware design, emphasizing robustness, transparency, and clinical validation. Looking forward, the most impactful advances are likely to arise from multimodal foundation models that integrate histopathology with molecular, clinical, and radiologic data, as well as from interactive pathologist-in-the-loop systems that augment rather than replace human expertise. Standardized multi-institutional benchmarking and prospective clinical studies will be essential to establish trust and demonstrate real-world value. Ultimately, the success of PFMs will depend on both their technical sophistication and their ability to integrate seamlessly into clinical workflows. Bridging this gap represents the central challenge and opportunity for the next generation of computational pathology systems.

## Figures and Tables

**Table 1:** Timeline of current gigapixel-scale foundation model development.

Year	Milestone	Result
2017	Transformers introduced	Basis for Vision Transformer (ViT) * and multimodal models
2020	Vision Transformer (ViT) *	First pure-attention image model
2021	Data-Efficient Image Transformers (DeiTs) * and Shifted Window Transformers (Swins) *	Data efficiency and scalability to whole-slide images (WSIs) *
2022	Masked Autoencoder (MAE) * and Segment Anything Model (SAM) *	Self-supervised pretraining for pathology and promptable segmentation
2023–2025	Vision–language foundation models (FMs) *	Gigapixel-scale foundation models (UNI, GigaPath, and Mayo Atlas)

* DeiT, Data-Efficient Image Transformer; FM, foundation model; MAE, Masked Autoencoder; SAM, Segment Anything Model; Swin, Shifted Window Transformer; ViT, Vision Transformer; WSI, whole-slide image.

**Table 2:** Chronological development of PFMs from academia and industry.

Model	Year	Type	Training Strategy	Data Scale	Domains	Key Contribution	Clinical Maturity
CLAM * [43]	2021	Model	Weakly supervised MIL	TCGA * + institutional cohorts	Multiorgan	Attention-based MIL *; key clinical precursor to PFMs	Research
HIPT * [21]	2022	Model	Hierarchical Vision Transformer	TCGA + public WSIs	Pan-cancer	First practical hierarchical ViT * for gigapixel WSIs *	Research
**UNI** [26]	2023	FM *	Self-supervised contrastive ViT	Multi-cohort, millions of tiles	Multiorgan	Open, reusable pathology embeddings for diverse downstream tasks	Research/Preclinical
**Virchow** [29]	2023	FM	Contrastive self-supervised ViT	>1 M WSIs *	Multiorgan	Robust cross-tissue morphologic representations at scale	Research/Preclinical
CONCH * [44]	2024	FM	Contrastive multimodal (vision–language)	>1 M WSIs with paired text	Multiorgan	Explicit multimodal PFM	Research
**GigaPath** [27]	2024	FM	Multi-task ViT-based pretraining	>1 M WSIs	Multiorgan	Enterprise-scale PFM emphasizing scalability	Research/Translational (enterprise-focused)
**Virchow 2** [45]	2025	FM	Expanded self-supervised ViT	>1 M WSIs, multi-institutional WSIs	Multiorgan	Improved scaling, robustness, and performance on rare and pan-cancer tasks	Research/Preclinical
TITAN * [31]	2025	FM	Self-supervised ViT	Large multi-institutional WSI cohorts	Multiorgan	Task-agnostic PFM emphasizing generalization	Research
**Mayo Clinic Atlas** [30]	2025	FM	Self-supervised ViT-H/14	~1.2 M WSIs	Multiorgan	Large curated institutional PFM with broad benchmark evaluation	Research/Translational (enterprise-focused)
**Phikon** [28]	2023	FM	Self-supervised ViT (DINOv2-based)	460 M patches extracted from 55 thousand slides	Multiorgan	Open-access pathology encoder optimized for general-purpose feature extraction and downstream adaptability	Research/Preclinical
**H-Optimus** [46]	2024	FM	Self-supervised hierarchical ViT (WSI-scale pretraining)	1 M WSIS	Multiorgan	Hierarchical whole-slide-level modeling enabling improved context-aware representations across gigapixel images	Research/Preclinical

* CLAM, Clustering-Constrained Attention Multiple Instance Learning; CONCH, CONtrastive learning from Captions for Histopathology; FM, foundation model; HIPT, Hierarchical Image Pyramid Transformer; M, million; MIL, Multiple Instance Learning; TCGA, The Cancer Genome Atlas; TITAN, Transformer-Based Pathology Image and Text Alignment Network; ViT, Vision Transformer; WSI, whole-slide image.

## Data Availability

No new data were created or analyzed in this study. Data sharing is not applicable to this article.

## References

[1] Vaswani A., Shazeer N., Parmar N., Uszkoreit J., Jones L., Gomez A.N., Kaiser L., Polosukhin I. (2017). Attention Is All You Need. http://arxiv.org/abs/1706.03762.

[2] Mancas M., Ferrera V.P., Coutrot A. (2025). From Human Attention to Computational Attention: A Multidisciplinary Approach.

[3] Yu R.T.-Y., Picard C., Ahmed F. (2025). Fast and accurate Bayesian optimization with pre-trained transformers for constrained engineering problems. Struct. Multidiscip. Optim..

[4] Liu Y., Zhang Y., Wang Y., Hou F., Yuan J., Tian J., Zhang Y., Shi Z., Fan J., He Z. (2024). A Survey of Visual Transformers. IEEE Trans. Neural Netw. Learn. Syst..

[5] Xu H., Xu Q., Cong F., Kang J., Han C., Liu Z., Madabhushi A., Lu C. (2024). Vision Transformers for Computational Histopathology. IEEE Rev. Biomed. Eng..

[6] Li Z., Cong Y., Chen X., Qi J., Sun J., Yan T., Yang H., Liu J., Lu E., Wang L. (2023). Vision transformer-based weakly supervised histopathological image analysis of primary brain tumors. iScience.

[7] Chaurasia A.K., Harris H.C., Toohey P.W., Hewitt A.W. (2025). A generalised vision transformer-based self-supervised model for diagnosing and grading prostate cancer using histological images. Prostate Cancer Prostatic Dis..

[8] He K., Zhang X., Ren S., Sun J. (2015). Deep Residual Learning for Image Recognition. http://arxiv.org/abs/1512.03385.

[9] Huang G., Liu Z., van der Maaten L., Weinberger K.Q. (2016). Densely Connected Convolutional Networks. http://arxiv.org/abs/1608.06993.

[10] Ronneberger O., Fischer P., Brox T., Navab N., Hornegger J., Wells W.M., Frangi A.F. (2015). U-Net: Convolutional Networks for Biomedical Image Segmentation. Medical Image Computing and Computer-Assisted Intervention—MICCAI 2015.

[11] Riasatian A., Babaie M., Maleki D., Kalra S., Valipour M., Hemati S., Zaveri M., Safarpoor A., Shafiei S., Afshari M. (2021). Fine-Tuning and training of densenet for histopathology image representation using TCGA diagnostic slides. Med. Image Anal..

[12] Ström P., Kartasalo K., Olsson H., Solorzano L., Delahunt B., Berney D.M., Bostwick D.G., Evans A.J., Grignon D.J., Humphrey P.A. (2020). Artificial intelligence for diagnosis and grading of prostate cancer in biopsies: A population-based, diagnostic study. Lancet Oncol..

[13] Madabhushi A., Feldman M.D., Leo P. (2020). Deep-learning approaches for Gleason grading of prostate biopsies. Lancet Oncol..

[14] Hamida A.B., Devanne M., Weber J., Truntzer C., Derangère V., Ghiringhelli F., Forestier G., Wemmert C. (2021). Deep learning for colon cancer histopathological images analysis. Comput. Biol. Med..

[15] Davri A., Birbas E., Kanavos T., Ntritsos G., Giannakeas N., Tzallas A.T., Batistatou A. (2022). Deep Learning on Histopathological Images for Colorectal Cancer Diagnosis: A Systematic Review. Diagnostics.

[16] Davri A., Birbas E., Kanavos T., Ntritsos G., Giannakeas N., Tzallas A.T., Batistatou A. (2023). Deep Learning for Lung Cancer Diagnosis, Prognosis and Prediction Using Histological and Cytological Images: A Systematic Review. Cancers.

[17] Touvron H., Cord M., Douze M., Massa F., Sablayrolles A., Jégou H. (2020). Training Data-Efficient Image Transformers & Distillation Through Attention. http://arxiv.org/abs/2012.12877.

[18] Liu Z., Lin Y., Cao Y., Hu H., Wei Y., Zhang Z., Lin S., Guo B. (2021). Swin Transformer: Hierarchical Vision Transformer using Shifted Windows. http://arxiv.org/abs/2103.14030.

[19] Kirillov A., Mintun E., Ravi N., Mao H., Rolland C., Gustafson L., Xiao T., Whitehead S., Berg A.C., Lo W.-Y. (2023). Segment Anything. http://arxiv.org/abs/2304.02643.

[20] Ravi N., Gabeur V., Hu Y.-T., Hu R., Ryali C., Ma T., Khedr H., Rädle R., Rolland C., Gustafson L. (2024). SAM 2: Segment Anything in Images and Videos. http://arxiv.org/abs/2408.00714.

[21] Chen R.J., Chen C., Li Y., Chen T.Y., Trister A.D., Krishnan R.G., Mahmood F. (2022). Scaling Vision Transformers to Gigapixel Images via Hierarchical Self-Supervised Learning. http://arxiv.org/abs/2206.02647.

[22] Tizhoosh H.R., Diamandis P., Campbell C.J., Safarpoor A., Kalra S., Maleki D., Riasatian A., Babaie M. (2021). Searching Images for Consensus: Can AI Remove Observer Variability in Pathology?. Am. J. Pathol..

[23] Kalra S., Tizhoosh H.R., Choi C., Shah S., Diamandis P., Campbell C.J., Pantanowitz L. (2020). Yottixel—An Image Search Engine for Large Archives of Histopathology Whole Slide Images. Med. Image Anal..

[24] Hegde N., Hipp J.D., Liu Y., Emmert-Buck M., Reif E., Smilkov D., Terry M., Cai C.J., Amin M.B., Mermel C.H. (2019). Similar image search for histopathology: SMILY. npj Digit. Med..

[25] Wang Y., Gu Y., Zhang X., Wang B., Wang R., Li X., Liu Y., Qu F., Ren F., Yan R. (2025). Computational pathology in precision oncology: Evolution from task-specific models to foundation models. Chin. Med. J..

[26] Chen R.J., Ding T., Lu M.Y., Williamson D.F.K., Jaume G., Song A.H., Chen B., Zhang A., Shao D., Shaban M. (2024). Towards a general-purpose foundation model for computational pathology. Nat. Med..

[27] Xu H., Usuyama N., Bagga J., Zhang S., Rao R., Naumann T., Wong C., Gero Z., González J., Gu Y. (2024). A whole-slide foundation model for digital pathology from real-world data. Nature.

[28] Filiot A., Jacob P., Kain A.M., Saillard C. (2024). Phikon-v2, a Large and Public Feature Extractor for Biomarker Prediction. http://arxiv.org/abs/2409.09173.

[29] Vorontsov E., Bozkurt A., Casson A., Shaikovski G., Zelechowski M., Severson K., Zimmermann E., Hall J., Tenenholtz N., Fusi N. (2024). A foundation model for clinical-grade computational pathology and rare cancers detection. Nat. Med..

[30] Alber M., Tietz S., Dippel J., Milbich T., Lesort T., Korfiatis P., Krügener M., Cancer B.P., Shah N., Möllers A. (2025). Atlas: A Novel Pathology Foundation Model by Mayo Clinic, Charit’e, and Aignostics. http://arxiv.org/abs/2501.05409.

[31] Ding T., Wagner S.J., Song A.H., Chen R.J., Lu M.Y., Zhang A., Vaidya A.J., Jaume G., Shaban M., Kim A. (2025). A multimodal whole-slide foundation model for pathology. Nat. Med..

[32] Pisapia P., L’Imperio V., Galuppini F., Sajjadi E., Russo A., Cerbelli B., Fraggetta F., d’Amati G., Troncone G., Fassan M. (2022). The evolving landscape of anatomic pathology. Crit. Rev. Oncol. Hematol..

[33] Mettman D.J., Gao L., Evans K., Frey A.B., Scheuner M.T., Klutts J.S., Frias-Kletecka M.C., Wang-Rodriguez J., Becker D.J., Mathur S.C. (2025). Mapping Pathology Work Associated with Precision Oncology Testing. Fed. Pract..

[34] Walsh E., Orsi N.M. (2024). The current troubled state of the global pathology workforce: A concise review. Diagn. Pathol..

[35] Humphrey P.A. (2010). Diagnostic anatomic pathology in the era of molecular medicine. Mo. Med..

[36] Wick M.R., Nappi O., Pfeifer J.D. (2013). Molecular techniques in anatomic pathology: An overview. Semin. Diagn. Pathol..

[37] Hunt J.L. (2017). Applications of molecular testing in surgical pathology of the head and neck. Mod. Pathol..

[38] VanderLaan P.A., Roy-Chowdhuri S., Griffith C.C., Weiss V.L., Booth C.N. (2022). Molecular testing of cytology specimens: Overview of assay selection with focus on lung, salivary gland, and thyroid testing. J. Am. Soc. Cytopathol..

[39] Prat J. (2017). Pathology of borderline and invasive cancers. Best Pract. Res. Clin. Obstet. Gynaecol..

[40] Verghese G., Lennerz J.K., Ruta D., Ng W., Thavaraj S., Siziopikou K.P., Naidoo T., Rane S., Salgado R., Pinder S.E. (2023). Computational pathology in cancer diagnosis, prognosis, and prediction—Present day and prospects. J. Pathol..

[41] Ochi M., Komura D., Ishikawa S. (2025). Pathology Foundation Models. JMA J..

[42] Campanella G., Chen S., Singh M., Verma R., Muehlstedt S., Zeng J., Stock A., Croken M., Veremis B., Elmas A. (2025). A clinical benchmark of public self-supervised pathology foundation models. Nat. Commun..

[43] Lu M.Y., Williamson D.F.K., Chen T.Y., Chen R.J., Barbieri M., Mahmood F. (2021). Data-efficient and weakly supervised computational pathology on whole-slide images. Nat. Biomed. Eng..

[44] Lu M.Y., Chen B., Williamson D.F.K., Chen R.J., Liang I., Ding T., Jaume G., Odintsov I., Le L.P., Gerber G. (2024). A visual-language foundation model for computational pathology. Nat. Med..

[45] Zimmermann E., Vorontsov E., Viret J., Casson A., Zelechowski M., Shaikovski G., Tenenholtz N., Hall J., Klimstra D., Yousfi R. (2024). Virchow2: Scaling Self-Supervised Mixed Magnification Models in Pathology. http://arxiv.org/abs/2408.00738.

[46] Bioptimus/H-optimus-1 Hugging Face. https://huggingface.co/bioptimus/H-optimus-1.

[47] Zhang A., Jaume G., Vaidya A., Ding T., Mahmood F. (2025). Accelerating Data Processing and Benchmarking of AI Models for Pathology. http://arxiv.org/abs/2502.06750.

[48] Bilal M., Gulzar M.A., Jaffar N., Albduljabbar A., Altherwy Y., Alsuhaibani A., Almarshad F. (2026). Benchmarking pathology foundation models for predicting microsatellite instability in colorectal cancer histopathology. Comput. Med. Imaging Graph..

[49] Neidlinger P., El Nahhas O.S.M., Muti H.S., Lenz T., Hoffmeister M., Brenner H., van Treeck M., Langer R., Dislich B., Behrens H.M. (2025). Benchmarking foundation models as feature extractors for weakly supervised computational pathology. Nat. Biomed. Eng..

[50] Ma J., Guo Z., Zhou F., Wang Y., Xu Y., Li J., Yan F., Cai Y., Zhu Z., Jin C. (2026). A generalizable pathology foundation model using a unified knowledge distillation pretraining framework. Nat. Biomed. Eng..

[51] Campanella G., Kumar N., Nanda S., Singi S., Fluder E., Kwan R., Muehlstedt S., Pfarr N., Schüffler P.J., Häggström I. (2025). Real-world deployment of a fine-tuned pathology foundation model for lung cancer biomarker detection. Nat. Med..

[52] de Jong E.D., Marcus E., Teuwen J. (2025). Current Pathology Foundation Models Are Unrobust to Medical Center Differences. http://arxiv.org/abs/2501.18055.

[53] Website. https://www.cancer.gov/tcga.

[54] Chen R.J., Wang J.J., Williamson D.F.K., Chen T.Y., Lipkova J., Lu M.Y., Sahai S. (2023). Algorithmic fairness in artificial intelligence for medicine and healthcare. Nat. Biomed. Eng..

[55] Website. https://camelyon16.grand-challenge.org/Data/.

[56] Montezuma D., Porz R., Ameisen D., L’Imperio V., Serbanescu M.S., Temprana-Salvador J., Zerbe N., Khalili N., Zlobec I., European Society of Digital and Integrative Pathology (ESDIP) (2025). Unbiased Artificial Intelligence: Addressing Bias in Computational Pathology. Mayo Clin. Proc. Digit. Health.

[57] Ma J., Xu Y., Zhou F., Wang Y., Jin C., Guo Z., Wu J., Tang O.K., Zhou H., Wang X. (2025). PathBench: A Comprehensive Comparison Benchmark for Pathology Foundation Models Towards Precision Oncology. http://arxiv.org/abs/2505.20202.

[58] Hays P. (2024). Artificial intelligence in cytopathological applications for cancer: A review of accuracy and analytic validity. Eur. J. Med. Res..

[59] Kim D., Thrall M.J., Michelow P., Schmitt F.C., Vielh P.R., Siddiqui M.T., Sundling K.E., Virk R., Alperstein S., Bui M.M. (2024). The current state of digital cytology and artificial intelligence (AI): Global survey results from the American Society of Cytopathology Digital Cytology Task Force. J. Am. Soc. Cytopathol..

[60] Reis-Filho J.S., Kather J.N. (2023). Overcoming the challenges to implementation of artificial intelligence in pathology. J. Natl. Cancer Inst..

[61] Abdul Razak M.S., Nirmala C.R., Sreenivasa B.R., Lahza H., Lahza H.F.M. (2022). A survey on detecting healthcare concept drift in AI/ML models from a finance perspective. Front. Artif. Intell..

[62] Kore A., Bavil E.A., Subasri V., Abdalla M., Fine B., Dolatabadi E. (2024). Empirical data drift detection experiments on real-world medical imaging data. Nat. Commun..

[63] Ivezić V., Radhachandran A., Redekop E., Athreya S., Lee D., Sant V., Arnold C., Speier W. (2025). CytoFM: The First Cytology Foundation Model. http://arxiv.org/abs/2504.13402.

[64] Huang Y., Zhao W., Zhang Z., Chen Y., Fu Y., Wu F., Jiang Y., Liang L., Wang S. (2025). Knowledge-guided adaptation of pathology foundation models effectively improves cross-domain generalization and demographic fairness. Nat. Commun..

[65] Lee J., Lim J., Byeon K., Kwak J.T. (2025). Benchmarking pathology foundation models: Adaptation strategies and scenarios. Comput. Biol. Med..

[66] Pescia C., Sozanska A.M., Thomas E., Cooper R.A. (2025). Artificial intelligence in haematopathology: Current perspective and future directions. Diagn. Histopathol..

[67] Chong Y., Fernández Aceñero M.J., Li Z., Bychkov A. (2026). Integration of Digital Cytology in Quality Assurance Programs for Cytopathology. Acta Cytol..

[68] Jung C.K., Kim C., Jeon S., Bychkov A. (2025). Quantitative Assessment of Focus Quality in Whole-Slide Imaging of Thyroid Liquid-Based Cytology Using Laplacian Variance. Endocr. Pathol..

[69] VandeHaar M.A., Al-Asi H., Doganay F., Yilmaz I., Alazab H., Xiao Y., Balan J., Dangott B.J., Nassar A., Reynolds J.P. (2025). Challenges and Opportunities in Cytopathology Artificial Intelligence. Bioengineering.

[70] Shean R.C., Rets A.V. (2026). Digital Pathology in Hematopathology: From Vision to Deployment. Int. J. Lab. Hematol..

[71] Giansanti D. (2024). AI in Cytopathology: A Narrative Umbrella Review on Innovations, Challenges, and Future Directions. J. Clin. Med..

[72] Rau T.T., Cross W., Lastra R.R., Lo R.C.-L., Matoso A., Herrington C.S. (2024). Closing the loop—The role of pathologists in digital and computational pathology research. J. Pathol. Clin. Res..

[73] Farris A.B., van der Laak J., van Midden D. (2025). Artificial intelligence-enhanced interpretation of kidney transplant biopsy: Focus on rejection. Curr. Opin. Organ Transplant..

[74] Park J.Y., Kim J., Kim Y.J., Kim S.H., An C.S., Kim K.G., Jung C.K. (2025). Multi-institutional validation of AI models for classifying urothelial neoplasms in digital pathology. Sci. Rep..

